# Quality assessment in sickness certificates – changes over an eight-year period in Sweden and associated factors

**DOI:** 10.1080/02813432.2025.2577668

**Published:** 2025-10-28

**Authors:** Magdalena Fresk, Wilhelmus J. A. Grooten, Lars G. Backlund, Britt Arrelöv, Ylva Skånér, Peter Henriksson, Anna Kiessling

**Affiliations:** a Department of Neurobiology, Care Sciences and Society, Division of Family Medicine and Primary Care, Karolinska Institutet, Huddinge, Sweden; b Department of Neurobiology, Care Sciences and Society, Division of Physiotherapy, Karolinska Institutet, Huddinge, Sweden; c Women’s Health and Allied Health Professionals Theme, Medical Unit Occupational Therapy and Physiotherapy, Karolinska University Hospital, Stockholm, Sweden; d Department of Clinical Sciences, Danderyd Hospital, Karolinska Institutet, Stockholm, Sweden; e Department of Clinical Sciences, Danderyd Hospital, Stockholm, and Department of Medical Specialities, Danderyd University Hospital, Karolinska Institutet, Stockholm, Sweden

**Keywords:** Disability and health, insurance medicine, mental health, primary health care, sick leave, social security, work capacity evaluation

## Abstract

**Aims:**

This study investigates changes in the quality of information in sickness certificates over time, before and after the 2011 national introduction of a revised sickness certificate, and identified factors associated with certificate quality.

**Methods:**

Four experts independently assessed the quality of a total of 783 sickness certificates issued in primary care in 2004, 2009, and 2012. A Global Quality Score (GQS) was constructed by the research group, rating quality on a 10-point scale. The cut-off for high quality was set to GQS >5. The inter-rater reliability of the GQS was tested using Intra-Class Correlation and internal consistency by Pearson correlation coefficients (r). Sickness certificates issued in 2004 and 2009 were merged into one group and compared to sickness certificates in 2012. Logistic binomial regression analyses examined associations between patient-, sick leave-, and physician-related variables and GQS.

**Results:**

The GQS demonstrated moderate to good inter-rater reliability (ICC= 0.79, 95% CI: 0.6–0.9) and good internal consistency. Certificate quality improved significantly (*p* < 0.001) from 3.6 (SD 1.5) in 2004/2009 to 4.7 (SD 1.2) in 2012. In 2004/2009, mental disorder or an extended length of sick leave was negatively associated with high quality. In 2012, only female sex of the patient was negatively associated with high certificate quality.

**Conclusion:**

While sickness certificate quality was overall low, it improved significantly after the 2011 revision of the sickness certificate. Also, diagnosis, sick leave duration, and patient’s sex were factors associated with the quality and varied over time, highlighting the need for further research regarding potential remaining differences in quality of sickness certificates.

## Introduction

**Figure 1.**
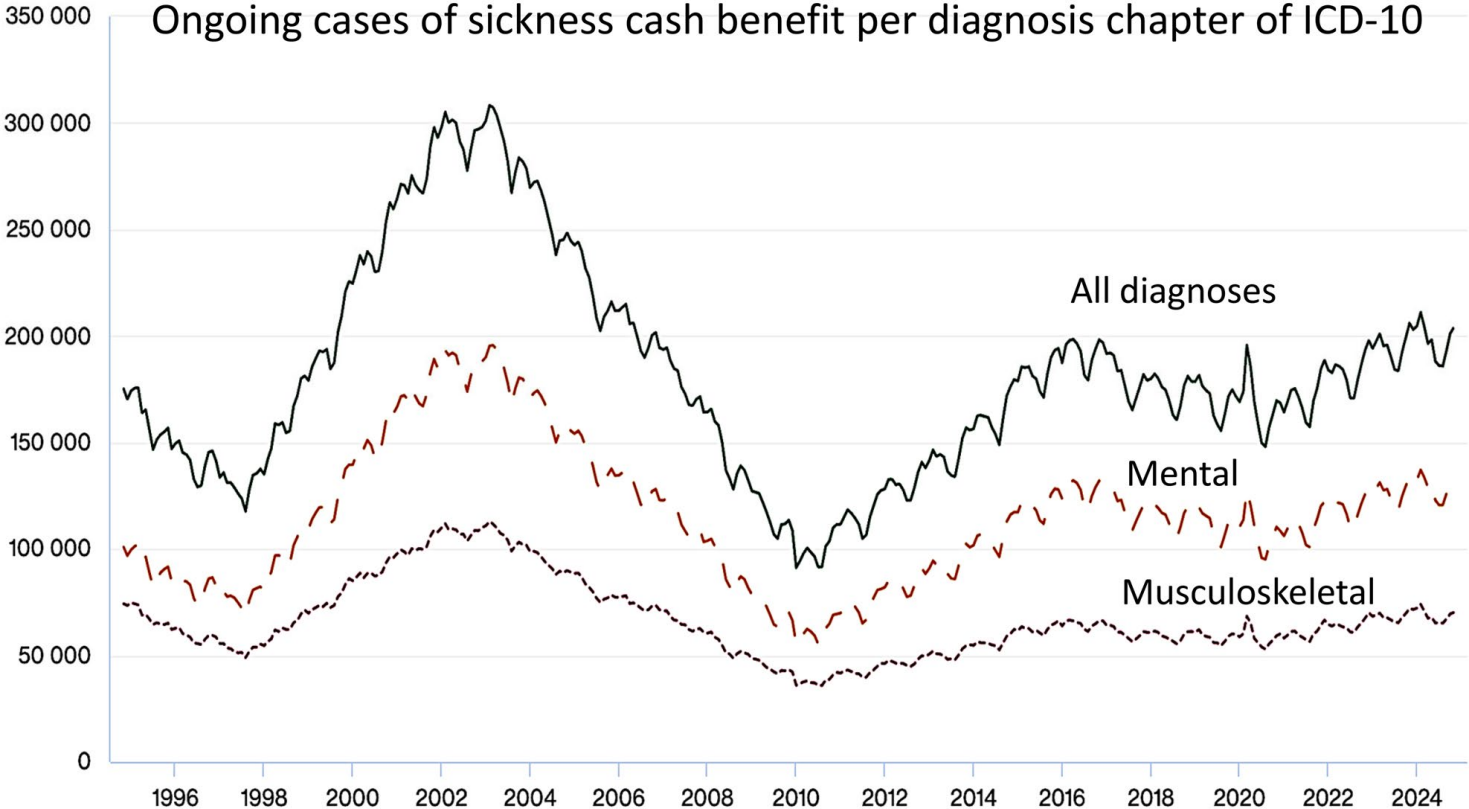
Ongoing cases of sickness benefit per ICD-10 diagnosis chapter (Swedish version). Figure adapted from: Swedish Social Insurance Agency Statistic database.

Nationwide cross-sectional questionnaire studies, as well as qualitative studies conducted in Sweden, have shown that sickness certification is perceived as problematic and complex by physicians [1–5]. In a systematic review of 56 studies, the vast majority from Scandinavia, Letrilliart et al. identified that the main challenges were related to work ability assessments, the doctor-patient relationship, the organization of the healthcare, and the socioeconomic context [6]. Similar difficulties exist across Europe, and various measures have been implemented at the national level to address these issues [7]. In Sweden, sick leave is managed by physicians of a wide range of medical specialties, but general practitioners seem to find it most problematic [4,8]. The proportion of mental disorders as the cause of sick leave has increased to 49.3% of ongoing cases of sickness cash benefit in December 2024 during the last two decades, followed by musculoskeletal disorders (17.8%) and other diseases (32.9%) [9,10] (Figure 1). Public expenditure on disability and sickness cash benefits is higher in Sweden compared to the OECD average [11].

The Swedish healthcare system and the Swedish Social Insurance Agency have distinct roles in the sickness certification process. Physicians assess the patient’s medical condition and work ability and issue sickness certificates, while the Social Insurance Agency determines eligibility for sickness benefits based on legal regulations [9,12]. High-quality information in sickness certificates is therefore essential from the perspectives of both the patient and other stakeholders.

In Sweden, strategies to reduce sickness absence over the past decades have included a broad range of measures aimed at improving sick-listing practices. National sick leave guidelines were introduced in 2007 to provide decision support for assessing work ability and determining the appropriate length of sick leave for the most common diagnoses [13]. These guidelines have been increasingly used by physicians over time [14]. In 2008, minor adjustments were made to the sickness certificate form to emphasize the need for information regarding work-related disability (Supplementary Appendices 1 and 2). In 2009, the Swedish government inquiry ‘*Borderland between Illness and Work’* emphasized that the WHO’s International Classification of Functioning, Disability and Health (ICF) could serve as a valuable foundation for assessing work ability in a more nuanced and standardized manner [15]. It could also support more transparent and equitable assessments of eligibility for sickness benefits. ICF was published by the World Health Organization (WHO) in 2001 and serves as a standardized framework for describing and organizing information on functioning and disability [16]. The classification considers a person’s health condition in relation to:

- Body functions and structures
- Activities and participation
- Environmental factors
- Personal factors (not yet coded)

As part of this development, in 2010, the Swedish Social Insurance Agency formally adopted an assessment model based on the ICF structure. This model requires that the disease leading to sick leave be described alongside the resulting impairments in body functions and activity limitations. In 2011, a major revision of the sickness certificate form was introduced to align with this model, requiring physicians to describe how the disease affects the patient’s functioning and ability to work (Supplementary Appendices 3 and 4). Since then, physicians have been required to describe functional impairments and activity limitations caused by the disease in each case of sick leave [13]. Another step in supporting the implementation of the ICF in sickness certification was taken in 2019, when an ICF-based documentation tool for recording impairments and activity limitations was introduced into the national digital sickness certificate form, where it remains in use [17].

Several studies have highlighted the inadequate quality of sickness certificates in Sweden. In 2005, Söderberg et al. reported that 73% of certificates from primary care lacked sufficient information to support decisions regarding sickness benefits [18]. In 2007, Nilsing et al. found that 35% of 475 certificates lacked necessary information on functioning [19]. Sturesson et al. reported that certificates issued in 2010–2011 for mental disorders often lacked details on activity limitations [20]. Similarly, Kiessling et al. noted that detailed certificates for long-term sickness absence issued in primary and secondary care in 2007 were generally of low quality and lacked the necessary information requested by the Swedish Social Insurance Agency [21]. The study found that certificates issued in primary care had lower quality compared to those issued in other areas of specialization, which could be explained by a different case mix in primary care [21]. Various methods have been used to assess the quality of information in sickness certificates. Qualitative analyses, some of them based on ICF, have been conducted to determine whether they contain sufficient information for decisions regarding sickness benefits [18–20,22]. Expert quality ratings have also been performed [21]. However, although there are reports indicating changes in the quality of sickness certificates over time, the methodological differences between existing studies limit the comparability of the results. In 2024, the Swedish National Board of Health and Welfare implemented follow-up measures to support healthcare regions in improving the effectiveness of sickness certification. Among the five prioritized areas, the quality of sickness certificates is emphasized as essential for ensuring accurate documentation in decisions regarding sickness benefits. These indicators aim to promote a more systematic and evidence-based approach, ultimately enhancing patient safety, efficiency, and quality of care [23]. However, there is currently no nationally or internationally established method for assessing the quality of sickness certificates. This lack of standardization presents challenges for consistent evaluation, research and improvement.

## Aims

This study aims to investigate changes in the quality of the information in sickness certificates before compared to after the introduction of a revised sickness certificate in 2011 and to identify factors associated with quality in sickness certificates.

## Materials and methods

### Study design

A cross-sectional study of three data collections of sickness certificates issued in primary care in a community in Stockholm County, Sweden over eight years.

### Setting

The study community is located northeast of Stockholm, Sweden. The characteristics of the population are similar to an average of Swedish communities and have been used in previous studies as a proxy for all Sweden [24]. The community had around 55,000 inhabitants during the study period, around 50% between 20 to 64 years of age. Approximately 10% were first-generation immigrants, the educational level was slightly lower than the national level and the median income was very close to average [25]. There were six primary healthcare centers and one local hospital.

### Data collection

**Figure 2.**
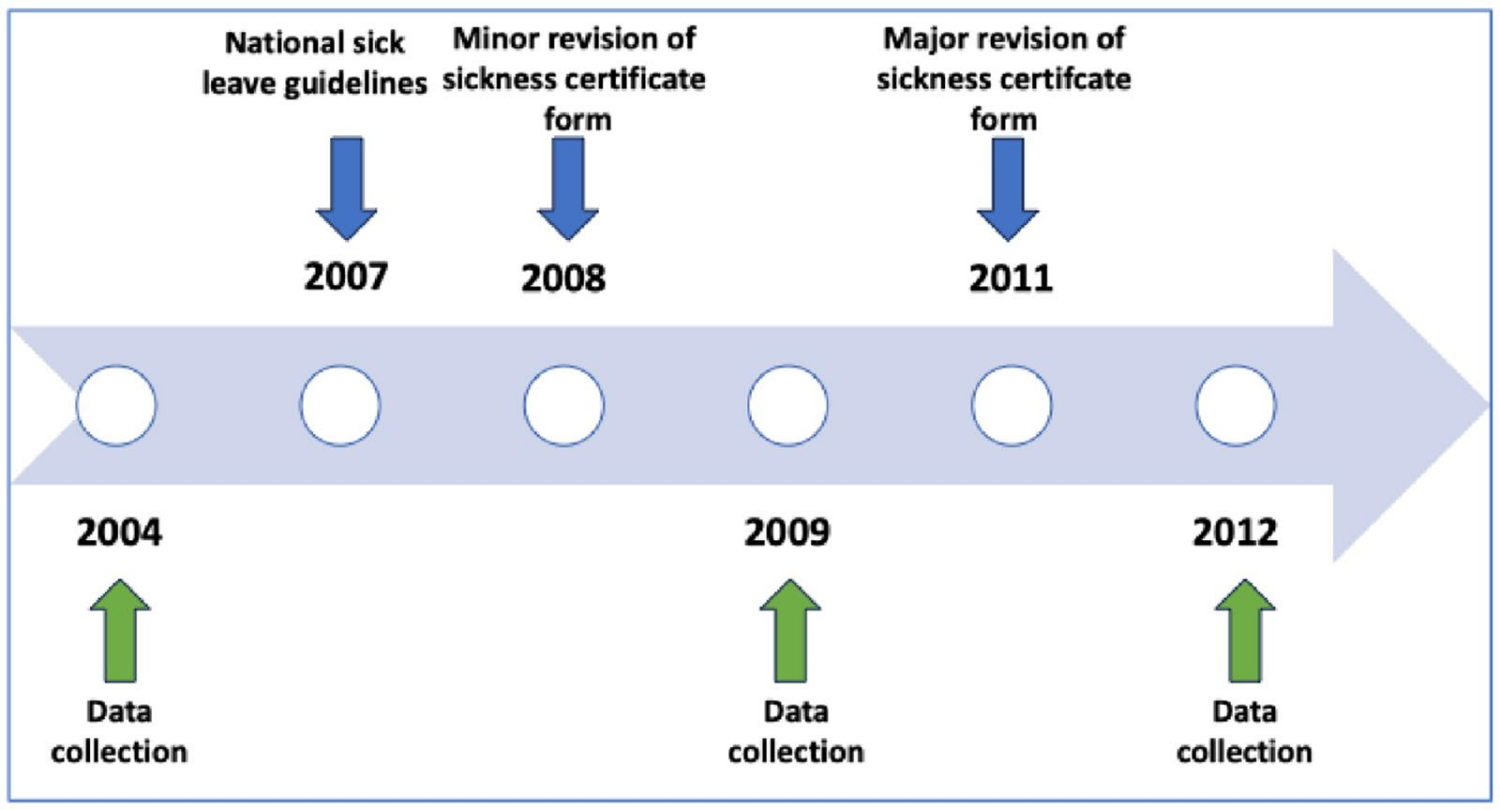
Illustration of a sample of national actions to support quality of sickness certificates in relation to the study’s data collection. The introduction of sick leave guidelines and revisions to the sickness certificate are marked above the timeline. Data collections of the study are marked below.

The data collection was conducted as part of a quality improvement initiative aimed at improving the sick leave certification process within Region Stockholm. Sickness certificates were collected during three separate periods: 2004/2005, 2009 and 2012 (Figure 2). Data was collected from the local Social Insurance Agency office. The second and third data collection periods were planned to match the first, but slight changes had to be made due to administrative changes at the Social Insurance Agency. Inclusion criteria were: 1) Sickness certificates registered at the local Social Insurance Agency during the study period, 2) Sickness certificates issued in primary care in the community, and 3) Sickness certificates concerning a sick leave episode of 14 days or longer. If more than one sickness certificate was issued for the same patient during a sickness period, only the most recent certificate was included. Identification data on the certificates were deleted before review. The study was approved by the regional ethical review board of Stockholm, Sweden; No. 04-794/5, 009/1344-31/5 and 2011/1872-31/5.

### Data collection 2004

All sickness certificates (*n* = 676) were consecutively collected at three strategic seasonal periods: in July 2004 and in February and May 2005. In total, 303 certificates were included based on inclusion criteria. The initial data collection was distributed across the calendar year to assess potential seasonal variation in diagnoses. As no such variation was observed, subsequent data collections were based on random sampling without consideration of season.

### Data collection 2009

A list of all sickness certificates in primary care registered at the local Social Insurance Agency from 1 January 2009 to 30 June 2009 was set up. Through randomization, 303 certificates that met the inclusion criteria were included in the study.

### Data collection 2012

A randomized sample of a total of 393 certificates registered at the local Social Insurance Agency and issued in the community from 1 January 2012 to 15 August 2012 were collected. Due to administrative changes at the Swedish Social Insurance Agency, the collected sample included certificates that had not been issued within primary care, and these were subsequently excluded. In total, 177 certificates fulfilled all inclusion criteria and were included in the study.

### Outcome variables

A Global Quality Score (GQS) was chosen as the primary outcome measure. The method for assessing the quality was designed by the research group, taking methods earlier used into account [21]. A quality assessment protocol was constructed and used in each assessment of the certificates (Supplementary Appendix 4). Each section of the sickness certificate was assessed, and as a final step, three global assessments were conducted: I) the extent to which the certificate included sufficient and relevant information, II) the extent to which the information provided was irrelevant or inconsistent, and III) Global Quality Score (GQS). The GQS was rated using a 10-point scale, where a score of 1 represented the lowest quality and 10 the highest. When assessing the GQS, the assessors should consider the overall relevance and completeness of the information in the sickness certificate. The GQS should also reflect whether all the information requested by the Social Insurance Agency was provided and if the information was clear and easy to understand. Each sickness certificate was assessed individually by all four assessors. Two assessors were also members of the research group; both were general practitioners with expertise in insurance medicine and extensive clinical experience in sickness certification. The other two assessors were experienced insurance officers at the Social Insurance Agency. Before the main assessments, a random sample of twenty certificates was assessed individually according to the protocol and then discussed in the group of four until consensus was achieved on how to perform the assessment of all sections and how to assess and interpret the GQS. These certificates were not included in the final study sample.

### Associated factors

Tentative factors associated with global quality were retrieved from the sickness certificates and grouped. Three patient-related variables were identified: the patient’s main diagnosis, age, and sex. Diagnoses were divided into three groups: ‘Mental’ (ICD-10 Chapter 5 – Mental and behavioral disorders); ‘Musculoskeletal’ (ICD-10 Chapter 7 – Diseases of the musculoskeletal system and connective tissue); and ‘Other,’ which included all other diagnoses [26]. Two sick leave-related variables were identified: ‘The degree of sick leave,’ categorized as either full-time sick leave or three different levels of part-time sick leave (25%, 50%, or 75% of full-time, dichotomized in the analyses as full-time or part-time). ‘The length of the sick leave period’ was expressed in days and categorized into four groups based on quartiles rounded up to integers: 1–14 days, 15–26 days, 27–42 days, and >42 days. In cases of part-time sick leave, all sick leave days were recalculated to the equivalent number of full-time sick leave days before the division into quartiles was performed. Finally, two physician-related variables were identified: physicians’ age and sex. It was not possible to distinguish between initial and renewed sick leave certificates based on the available data, and therefore this distinction could not be included in the analysis.

### Statistical analyses

For all variables, descriptive statistics were calculated. Continuous variables were expressed as mean and standard deviations (SD), and categorical variables were expressed as frequencies, percentages, or both unless otherwise indicated. The GQS of each certificate was calculated as the mean of the four experts′ ratings. The GQS of the certificates from 2004 and 2009 were merged into one group and were compared to the GQS of the certificates from 2012 using Student’s *t*-test. The level of significance was set to *p* < 0.05. The regression analyses on factors associated with high quality were divided into these two strata. The instrumental properties of the GQS had not previously been assessed. First, we tested whether there were systematic differences between the ratings of the four assessors using a one-way ANOVA. Second, inter-rater reliability for the GQS was calculated using the intraclass correlation. ICC estimates and their 95% confidence intervals were calculated, based on a single rating, absolute-agreement, 2-way mixed-effects model [27]. The 95% confident interval of the ICC estimate was the basis to evaluate the level of reliability: Values less than 0.5 are indicative of poor reliability, values between 0.5 and 0.75 indicate moderate reliability, values between 0.75 and 0.9 indicate good reliability, and values greater than 0.9 indicate excellent reliability [27]. Third, internal consistency was assessed by calculating Pearson correlations between the GQS and the experts’ ratings of: (1) the degree to which the certificate contained sufficient and relevant information, and (2) the degree to which the information was irrelevant or inconsistent [28]. The cut-off for significant good internal consistency was set to (*p* < 0.05) for both positive correlations between the GQS and sufficient and relevant information, as well as negative correlations between the GQS and irrelevant or inconsistent information.

Logistic binominal regression analyses explored the association between the patient-related, sickness absence-related, and physician-related variables (as independent variables) and the high global quality of the certificate (GQS > 5). Associations were expressed in odds ratios (ORs) with 95% confidence intervals (95%CI). Each variable was tested univariately, and those associated with the outcome at *p* < 0.1 were included in a multivariate initial regression model. The final regression model consisted of only significant variables (*p* < 0.05) from the initial model. These analyses were stratified for the merged certificates from 2004 and 2009, and for the certificates from 2012.

## Results

### Sickness certificates

A total of 783 certificates were included (Table 1). One certificate from 2004 had missing values on GQS (all raters) and was excluded from further statistical analysis. Another seven certificates from 2004 and eight from 2009 lacked GQS from at least one rater. These certificates had a mean GQS of 3.7, a value close to the mean GQS of the merged group of 2004 and 2009 (3.6, SD 1.5) and were included in the logistic regression. Moreover, a small percentage (0.5–6%) of the certificates had internal missing values, mainly on physician related variables (Table 1).

**Table 1.** Characteristics of all sickness certificates included in the study per data collection and in total.

	2004 (*n* = 303)		2009 (*n* = 303)		2012 (*n* = 177)		Total (*n* = 783)	
	n	%	n	%	n	%	n	%
**Patient age, year**								
Mean (SD)	45 (11)		45 (12)		48 (12)		46 (12)	
*Missing*	*0*		*1*		*0*		*1*	
**Patient sex **								
Male	106	35	102	34	48	27	256	33
Female	197	65	200	66	129	73	526	67
*Missing*	*0*		*1*		*0*		*1*	
**Main diagnosis **								
Mental	105	35	106	35	55	31	266	34
Musculoskeletal	118	39	97	32	58	33	274	35
Other	78	26	100	33	64	36	241	31
**Degree of sick leave **								
Full-time	210	70	210	69	124	70	544	70
Part-time	92	30	93	31	53	30	238	30
*Missing*	*1*		*0*		*0*		*1*	
**Length of sick leave, days **								
Mean (SD)	39 (28)		31(26)		32(30)		34(28)	
**Physician sex**								
Male	207	68	189	62	88	50	484	62
Female	95	31	84	28	78	44	257	33
*Missing*	*1*		*30*		*11*		*42*	
**Physician age, year**								
Mean (SD)	51 (10)		52 (12)		50 (12)		46 (12)	
*Missing*	*0*		*34*		*13*		*44*	

Missing reported for all variables where it occurs. Data given as number (%) of certificates if not otherwise noted. Age and length of sick leave in mean (SD).

### Measurement properties

The two Social Insurance Agency officers scored lower (mean (SD) GQS 3.2 (1.5) and 3.1 (1.5)) compared to the two physicians (mean (SD) 4.5 (2.5) and 4.7 (1.7), respectively (*p* < 0.001) (One-way ANOVA, *p* value < 0.001). However, the ICC of GQS was estimated to be 0.79 (95%CI 0.6–0.9), indicating good inter-rater reliability [27]. A strong positive correlation was found between GQS and ‘sufficient and relevant information’ (*r* = 0.833; *p* < 0.001), along with a smaller but still significant negative correlation between GQS and ‘irrelevant or inconsistent information’ (*r* = –0.176; *p* < 0.001), indicating that the internal consistency was good.

### Global quality of the sickness certificates

#### Mean Global Quality Score

The mean GQS of all certificates was 3.9 (SD 1.5). The mean GQS of the sickness certificates for each subcategory in each variable is described in Table 2. The mean of GQS was 2.9 (SD 1.9) in 2004, 4.4 (SD 1.4) in 2009 and 4.7 (SD 1.2) in 2012. The mean GQS for the period 2004 and 2009 merged was 3.6 (SD 1.5). The Student’s *t*-test revealed a statistically significant (*p* < 0.001) improvement in the mean global quality from 2004/2009 and the certificates from 2012.

**Table 2.** Global Quality Score (mean of the four assessors) of the total sample of sickness certificates (n = 783) by patient-, sick leave-, and physician-related characteristics.

	Number of sickness certificates		Global Quality Score
	n	%	mean, (SD)
**Patient age, years**			
≤35	174	22	4.0 (1.5)
36–50	307	39	3.8 (1.6)
≥51	298	38	3.9 (1.5)
**Patient sex**			
Male	256	32	4.0 (1.6)
Female	525	67	3.8 (1.5)
**Main diagnosis**			
Mental	266	34	3.6 (1.3)
Musculoskeletal	274	35	4.1 (1.6)
Other	240	31	4.0 (1.5)
**Degree of sick leave**			
Full-time	543	69	3.9 (1.5)
Part-time	238	30	3.8 (1.5)
**Length of sickness certificate, days**			
1–14	187	24	4.0 (1.5)
15–26	206	26	4.1 (1.6)
27–42	191	25	3.8 (1.5)
43–180	197	25	3.7 (1.5)
**Physician sex**			
Male	483	65	3.7 (1.5)
Female	257	35	4.1 (1.6)
**Physician age, years**			
≤35	75	10	4.5 (1.6)
36–50	223	30	3.7 (1.4)
≥51	438	60	3.8 (1.6)

### Defining high/low global quality score

The GQS using all sickness certificates (2004, 2009 and 2012) was explored, and the highest quartile was found to be a relevant cut-off for defining high quality (GQS > 5). For the strata 2004/2009, 21.9% (129/606) of the certificates exceeded the cut-off of GQS 5, while in 2012 the percentage doubled to 40.7% (72/177).

### Factors associated with a high global quality score of the sickness certificate

#### Sample 2004/2009

For the certificates from 2004 and 2009, three variables turned out to be associated with quality (*p* < 0.1) and were included in the initial model (Table 3). Mental disorders had a significant odds ratio below 1, indicating that the certificates issued for patients with mental disorders were associated with lower quality, compared to the certificates with musculoskeletal diagnosis. Certificates on male patients were associated (*p* < 0.1) with a higher global quality, while the length of sick leave was negatively associated with high quality, meaning that a sickness certificate issued for a longer period had lower quality compared to certificates issued for a shorter period.

**Table 3.** Univariate logistic regression model on the association between patient, sick leave, and physician-related data and high quality of sickness certificates in the merged data collection 2004/2009. Odds ratios and 95% confidence intervals.

Univariate model 2004/2009					
	Total	Low quality	High quality	Odds ratio (95%CI)	*p* value
**Patient age**					
Continuous data, mean (SD)		45 (12)	45 (12)	0.998 (0.980–1.016)	0.819
**Patient sex**					
Female (ref)	396	334	62	1.0	
Male	208	164	44	1.45 (0.941–2.220)	0.093
**Diagnosis**					
Musculoskeletal (ref)	**215**	**163**	**52**	**1.0**	**0.001**
Mental	211	190	21	0.346 (0.200–0.599)	0.000
Other	177	144	33	0.718 (0.440–1.173)	0.186
**Part-time sick leave**					
No (ref)	419	348	71	1.0	
Yes	185	150	35	1.14 (0.731–1.790)	0.557
**Length of sick leave**					
1–14 days (ref)	**136**	**105**	**31**	**1.0**	**0.041**
15–26 days	156	123	33	0.91 (0.522–1.583)	0.735
27–42 days	**150**	**128**	**22**	**0.58 (0.318–1.065)**	**0.079**
> 42 days	**160**	**141**	**19**	**0.46 (0.244–0.852)**	**0.014**
**Physician – age**					
Continuous data, mean (SD)		52 (10)	52 (12)	0.999 (0.979–1.020)	0.932
**Physician – sex**					
Male (ref)	395	333	62	1.0	
Female	179	141	38	1.447 (0.924–2.268)	0.107

Values presented in bold indicate statistical significance (*p* < 0.05).

In the final model for 2004/2009, only two factors remained negatively associated with the high quality of the certificate: Mental disorders OR = 0.310 (95%CI 0.176–0.546) and a longer period of sick leave: 27–42 days: OR = 0.507 (95%CI 0.270–0.951) and >42 days:

OR = 0.427 (95%CI 0.224–0.816) (Table 4).

**Table 4.** Initial and final models of the logistic regression analyses for the association between patient-, sick leave-, and physician-related factors and high-quality of sickness certificates from 2004/2009. Odds ratios and 95% confidence intervals.

	Initial model 2004/2009		Final model 2004/2009	
	Odds ratio (95%CI)	*p* value	Odds ratio (95%CI)	*p* value
**Patient sex**				
Female (ref)	1.0			
Male	1.379 (0.885–2.148)	0.155		
**Diagnosis**				
Musculoskeletal (ref)	1.0	0.0003		0.0002
Mental illness	**0.318 (0.181**–**0.561)**	**0.000**	**0.310 (0.176**–**0.546)**	**0.000**
Other	0.599 (0.358–1.001)	0.050	0.606 (0.363–1.010)	0.055
**Length of sick leave**				
1–14 days	**1.0**	**0.017**		**0.020**
15–26 days	0.878 (0.495–1.556)	0.655	0.895 (0.506–1.584)	0.703
27–42 days	**0.494 (0.262**–**0.929)**	**0.029**	**0.507 (0.270**–**0.951)**	**0.034**
> 42 days	**0.416 (0.218**–**0.796)**	**0.008**	**0.427 (0.224**–**0.816)**	**0.010**

Values presented in bold indicate statistical significance (*p* < 0.05).

### Sample 2012

For the 2012 initial model, only the patient’s sex was significantly (*p* = 0.013) associated with a higher quality of the certificate, and certificates of female patients had a lower OR compared to male patients; OR = 0.421 (95%CI 0.212–0.834) (Table 5).

**Table 5.** Univariate logistic regression analyses for the association between patient-, sick leave-, and physician-related factors and high quality of sickness certificates from 2012. Odds ratios and 95% confidence intervals.

	Univariate model 2012						
	Total	Low quality	High quality	Odds ratio (95%CI)			*p* value
**Patient age. years**							
Continuous data, mean (SD)		47 (12)	48 (12)	1.005 (0.979–1.031)			0.701
Total	177	118	59				
**Patient sex**							
Male (ref)	48	25	23	1.0			
Female	**129**	**93**	**36**	**0.421 (0.212**–**0.834)**			**0.013**
Total	177	118	59				
**Diagnosis**							
Musculoskeletal (ref)	59	37	22	1.0			0.500
Mental illness	55	40	15	0.631 (0.285–1.395)			0.255
Other	63	41	22	0.902 (0.431–1.890)			0.786
Total	177	118	59				
**Part-time sick leave**							
No (ref)	124	78	46	1.0			
Yes	53	40	13	0.551 (0.267–1.137)			0.107
Total	177	118	59				
**Length of sick leave**							
1–14 days (ref)	51	40	11	1.0			0.119
15–26 days	50	28	22	2.857 (1.197–6.820)			0.018
27–42 days	41	28	13	1.688 (0.661–4.309)			0.273
>42 days	35	22	13	2.149 (0.825–5.594)			0.117
Total	177	118	59				
**Physician age. years**							
Continuous data, mean (SD)		51 (10)	49 (14)	0.984 (0.957–1.012)			0.268
Total	164	110	54				
**Physician sex**							
Male	88	61	27	1.0			
Female	78	50	28	1.265 (0.662–2.417)			0.476
Total	166	111	55				

Values presented in bold indicate statistical significance (*p* < 0.05).

## Discussion

Although the overall quality of the sickness certificates was low, the scores improved between 2004/2009 and 2012. Three factors were associated with quality, but the factors differed over time. For the sample of 2004/2009, certificates issued for patients with mental disorders and certificates issued for a longer sick leave period (>27 days) were associated with poor quality. In 2012, sickness certificates issued for female patients were associated with lower quality.

The increase in overall quality coincides with the introduction of major changes in the sickness certificate regarding descriptions of functional impairments and activity limitations according to the ICF model [16]. Other explanations for the improvement are also possible. Given the design of the study, we are not able to fully answer questions on the causality of quality changes over time. It has been shown that the quality of sickness certification practice in primary care improved during 2004–2009, as evidenced by fewer certified days on the first certificate and a higher proportion of complete and adequate certificates [29]. Additionally, an increased amount of information regarding functional impairments and activity limitations was observed in certificates issued a year after the introduction of national sick leave guidelines in 2008, compared to those issued before [19,22].

The finding that certificates issued due to mental disorders had lower quality in 2004/2009 is not surprising. Difficulties in describing psychological aspects of functioning and disability in relation to work capacity have been reported by Nilsing et al. in a 2007 study of 475 sickness certificates from hospitals, occupational health services, and primary care [19]. In a later quality assessment study conducted in 2010/2011, Sturesson et al. found that certificates issued for mental disorders more frequently lacked adequate descriptions of activity limitations compared to certificates issued for other diagnoses [20]. In a study of a more comprehensive medical certificate for disability evaluation in long-term sickness absence, issued in primary care in 2007, it was found that the overall quality was generally low, but also that the musculoskeletal conditions had lower quality than mental disorders [21]. The National Audit Office reported in 2018 that physicians in primary care stated that the instructions for requested information in the certification sections—functional impairments and activity limitations—were imprecise and not suitable for mental disorders [30]. Also, the physicians had difficulties describing the link between diagnosis, functional impairments, and activity limitations. Another complexity, according to the report, is that sickness certification should be supported by objective findings, which might be hard to observe in mental disorders and then verified by the physician on the certificate [30]. We found that sick leave due to a mental disorder was negatively associated with high quality in certificates issued in 2004/2009, but not in certificates issued in 2012. Although it is reasonable to believe that difficulties might still exist, there seems to have been an improvement in physicians’ ability to explain functional impairment and activity limitation in this patient group, which might have been supported by the changed format of the sickness certificate in 2011.

A longer sick leave period was negatively associated with high certificate quality in 2004/2009. A possible explanation is that the first sick leave period is often short. If there is a need for renewal for a longer period, the physicians then tend to refer to previous certificates rather than reiterate the case history and status. In a retrospective database study of information from 263,441 sickness certificates issued in primary care, Skånér et al. found that the quality of sickness certification practice improved during 2004–2009 [29]. One possible explanation suggested was the development of tools within the electronic health record to support physicians in saving and reusing information. These tools may at least partly explain why the association with a long sick leave period was no longer present in our sample from 2012.

In 2012, there was an association between certificates issued for female patients and lower quality regardless of diagnosis. The qualitative study of the quality of sickness certificates, by Sturesson et al. found that sickness certificates issued for men less often needed supplementary information, and the link between the diagnosis and information regarding functioning was clearer [20]. On the other hand, Nilsing et al. found no difference in the amount of information regarding functioning between male and female patients in a similar study of the quality of sickness certificates [19].

## Methodological considerations

The strengths of the study are the large sample size, and the long time period studied. The sample of certificates was from a geographical area chosen to be representative of a medium sized, middle-income community in Sweden. The period was relatively long, which is important for external validity. The outcome measure has both advantages and disadvantages. The assessment protocol used was similar to a protocol that was previously tested and used to assess the quality of medical certificates for long-term sickness absence [21]. The inter-rater reliability of the Global Quality Score and the internal consistency with the two related global ratings was good. The purpose of the quality assessment was to evaluate quality against established requirements of the sickness certification process, grounded in legal demands in both the healthcare and the social insurance system. Such an evaluation presupposes specific competence in insurance medicine and knowledge of the quality criteria, which is not necessarily part of routine clinical practice. For this reason, it is a strength of the study that two raters were experienced GPs with expertise in sickness certification, and two were senior officers from the Social Insurance Agency. This composition was intended to combine clinical and administrative perspectives and ensure that ratings reflected the standards applied in decision-making processes rather than individual clinical habits. Prior to the main assessment, the raters undertook a calibration exercise, and all certificates were then rated independently and blinded.

Our data is limited to the information given in the sickness certificates, and aspects such as contextual or competence-related factors were not accessible in this study. The study period (2004–2012) lies considerably in the past, and some adjustments to the sickness certificate have since then been implemented. Consequently, the results may not accurately reflect the current situation. However, the core structure of the sickness certificate and the requirements of the Swedish sickness certification process—particularly regarding the documentation of diagnosis, functional impairments, and activity limitations—have remained unchanged since the 2011 revision. As such, the findings of this study retain substantial relevance for the evaluation and improvement of current practices. Furthermore, the quality of the information in the sickness certificate may depend on earlier steps in the process, such as other parts of the work ability assessment, which were not covered in our study. Finally, the smaller number of certificates in the 2012 sample compared to the 2004/2009 samples could have decreased the possibility of finding significant associations due to a larger 95% CI, e.g. those factors associated with the 2004/2009 samples. However, the point estimates of the factors were increased, and the lack of associations does not seem to be a power problem.

## Implications

In this study, we assessed the overall quality of sickness certificates issued in 2004, 2009 and 2012. The results contribute to the knowledge on the quality of sickness certificates, a crucial component of the sickness certification process. The findings are relevant in light of ongoing national efforts to improve the quality and standardization of medical documentation. By employing a structured assessment method, the study not only provides a methodological foundation for future research but also offers a tool that can be applied in forthcoming studies to evaluate changes over time. In this way, the study supports the continued development of evidence-based practices in this field. To identify effective strategies for improving certificate quality, future research could include studies focusing more specifically on the content of physicians’ assessments of reduced work capacity. One approach could be to map the physicians’ descriptions to a standardized terminology such as the International Classification of Functioning, Disability and Health (ICF), thereby facilitating the production of comparable and systematically interpretable data.

## Conclusions

This study revealed that the overall quality of sickness certificates was initially low but has improved over time. The introduction of a revised sickness certificate in 2011 contributed to a more comprehensive and interpretable content in the certificates issued in 2012 compared to those from 2004/2009. The factors associated with certificate quality evolved throughout the study period, underscoring successful efforts to enhance the quality of certificates for patients with mental disorders. Nevertheless, there remains a need for further research regarding potential remaining differences in quality of sickness certificates, especially due to the patient′s sex.

## Supplementary Material

Appendix 3 Sickness_certificate_2012.pdf

Appendix 1_Sickness certificate 2004.pdf

Appendix_4_quality_assessement_protocol_resubmission_1.pdf

Appendix 2_Sickness Certificate 2009.pdf
