# Supplementary material for: Quality assessment in sickness certificates – changes over an eight-year period in Sweden and associated factors
Source: Scand J Prim Health Care. 2025 Oct 28;44(1):1–13. doi: 10.1080/02813432.2025.2577668 (PMC12918320; doi:10.1080/02813432.2025.2577668)
Supplement: Appendix 3 Sickness_certificate_2012.pdf [file IPRI_A_2577668_SM0706.pdf]

Appendix 3 Sickness Certiticate 2012

Försäkringskassan

Läkarintyg

enligt 27 kap. 25 § socialförsäkringsbalken

Patientens namn

Personnummer

Skickas till

Försäkringskassans inläsningscentral

839 88 Östersund

Om patienten inte är känd ska han eller hon styrka sin identitet genom legitimation med foto (SOSFS 2005:29)

1

☐ Avstängning enligt smittskyddslagen på grund av smitta (fortsätt till fält 8 b)

2 Diagnos/diagnoser för sjukdom som orsakar nedsatt arbetsförmåga

Diagnosis/diagnoses for health condition causing limitations in work ability*

Diagnoskod enligt ICD-10 (Huvuddiagnos)

Minst tre positioner

3 Aktuellt sjukdomsförlopp

4 Funktionsnedsättning - observationer, undersökningsfynd och utredningsresultat

Impairments in functioning - observations, findings, and results of investigations

Intyget baseras på

Datum

☐ min undersökning av patienten ____

☐ min telefonkontakt med patienten ____

☐ journaluppgifter ____

☐ annat (ange vad i fält 13) ____

72631103

5 Aktivitetsbegränsning relaterat till diagnos (fält 2) och funktionsnedsättning (fält 4)

Activity limitations in relation to diagnosis (section 2), and impairments in functioning (section 4)

6 a Rekommendationer

☐ kontakt med Arbetsförmedlingen

☐ kontakt med företagshälsovården

☐ övrigt (ange vad) ____

6 b Planerad eller pågående behandling eller åtgärd

☐ inom sjukvården (ange vilken) ____

☐ annan åtgärd (ange vilken) ____

Note: English translation by the author. Form is published with consent from the Swedish Social Insurance Agency.

FK7263 (009 L 001) Fastställd av Försäkringskassan i samråd med Socialstyrelsen 2010-04-13

7 Är arbetslivsinriktad rehabilitering aktuell?

☐ Ja ☐ Nej ☐ Går inte att bedöma

8 a Patientens arbetsförmåga bedöms i förhållande till

☐ nuvarande arbete - ange aktuella arbetsuppgifter

☐ arbetslöshet - att utföra sådant arbete som är normalt förekommande på arbetsmarknaden

☐ föräldraledighet med föräldrapenning - att vårda sitt barn

8 b Jag bedömer att patientens arbetsförmåga är

	från och med (år, månad, dag)	längst till och med (år, månad, dag)
☐ nedsatt med 1/4		
☐ nedsatt med hälften		
☐ nedsatt med 3/4		
☐ helt nedsatt		

9 Patientens arbetsförmåga bedöms nedsatt längre tid än den som det försäkringsmedicinska beslutsstödet anger, därför att:

10 Prognos - kommer patienten att få tillbaka sin arbetsförmåga i nuvarande arbete? (Gäller inte arbetslösa)

☐ Ja ☐ Ja, delvis ☐ Nej ☐ Går inte att bedöma (Förtydliga i fält 13)

11 Kan resor till och från arbetet med annat färdsätt än normalt göra det möjligt för patienten att återgå i arbete? ☐ Ja ☐ Nej

12 Kontakt önskas med Försäkringskassan ☐ Ja

13 Övriga upplysningar och förtydliganden

Ovanstående uppgifter och bedömningar bekräftas

14 Datum

15 Namnförtydligande, mottagningens adress och telefon

16 Läkarens namnteckning

17 Förskrivarkod och arbetsplatskod

72631203

FK7263 (009 L 001) Fastställd av Försäkringskassan i samråd med Socialstyrelsen 2010-04-13
