# Supplementary material for: Quality assessment in sickness certificates – changes over an eight-year period in Sweden and associated factors
Source: Scand J Prim Health Care. 2025 Oct 28;44(1):1–13. doi: 10.1080/02813432.2025.2577668 (PMC12918320; doi:10.1080/02813432.2025.2577668)
Supplement: Appendix 1_Sickness certificate 2004.pdf [file IPRI_A_2577668_SM0705.pdf]

Appendix 1 Sickness Cetificate 2004

Försäkringskassan

Klinik eller mottagning, tfnr och läkarens namn (om ej nedan)

MEDICINSKT UNDERLAG
- för bedömning av rätt till sjukpenning och eventuellt behov av rehabilitering

Patientens personnummer

Patientens namn

Läkarintyg enligt 3 kap. 8§ lagen om allmän försäkring.
Du kan även använda blanketten för avstängning enligt smittskyddslagen (SmL)

Om patienten inte är känd ska identiteten styrkas genom legitimationshandling med foto (SOSFS 1981:25)

1
☐ Avstängning enligt SmL på grund av smitta (fortsätt till punkt 8)

Medicinsk bedömning
Vid bedömningen ska du bortse från arbetsmarknadsmässiga, ekonomiska, sociala och liknande förhållanden.

2 Diagnos/-er eller symtom till grund för den nedsatta förmågan/aktivitetsbegränsningen

Diagnosis or symptoms causing the limitation in ability/activity

Diagnoskod enl ICD 10 (huvuddiagnos)
minst tre positioner

3 Anamnes (aktuell sjukdom)

Anamnesis (current disease)

4 Status, objektiva undersökningsfynd

Clinical status, objective findings

Uppgifterna baserade på | datum
☐ Personlig kontakt
☐ Telefonkontakt
☐ Journaluppgifter
☐ Annat (ange vad under punkt 13)

5 Hur begränsar sjukdomen patientens förmåga/aktivitet?

How does the disease limit the patient's ability/activity?

6 Föreskrift - behandling eller åtgärd som är nödvändig för att förmågan ska kunna återställas
☐ Följa given ordination (ange vilken)
☐ Fortsatt poliklinisk kontakt
☐ Undvika viss belastning (ange vilken)
☐ Besöka arbetsplatsen
☐ Väntar på åtgärd inom sjukvården (ange vilken)
☐ Väntar på annan åtgärd (ange vilken)
☐ Övrigt (ange vad)

72632101

RFV 7263 Formulär fastställt av RFV i samråd med Socialstyrelsen 03.06

Note: English translation by the author. Form is published with consent from the Swedish Social Insurance Agency.

7 Är arbetslivsinriktad rehabilitering aktuell?

☐ Ja ☐ Nej ☐ Kan inte bedömas för närvarande ☐ Behov av kontakt med företagshälsovård

8 Medicinsk bedömning av i vilken grad funktionsnedsättningen begränsar patientens förmåga

☐ att utföra sina vanliga arbetsuppgifter (ange arbetsuppgifternas art):

☐ om patienten är arbetslös; att söka/kunna utföra arbete som är normalt förekommande på arbetsmarknaden

☐ om patienten är föräldraledig med föräldrapenning; att vårda sitt barn

Arbetsförmågan bedöms

	fr.o.m. (år, mån, dag)	längst t.o.m. (år, mån, dag)
☐ delvis nedsatt med 1/4		
☐ delvis nedsatt med 1/2		
☐ delvis nedsatt med 3/4		
☐ helt nedsatt		

(om helt nedsatt, besvara frågorna nedan)

- Kan deltid vara olämplig av psykosociala skäl? ☐ Ja ☐ Nej
- Kan anpassade arbetsuppgifter möjliggöra sysselsättning på deltid/heltid? ☐ Ja ☐ Nej
- Kan deltid vara skadlig för sjukdomens förlopp? ☐ Ja ☐ Nej
- Kan deltid i nuvarande sysselsättning vara möjlig med hänsyn till symtom? ☐ Ja ☐ Nej
- Kan deltid förbättra prognosen för återgång i arbete? ☐ Ja ☐ Nej
- Kan deltidsarbete på annat sätt vara skadligt? ☐ Ja ☐ Nej

9 Prognos - bedöms patienten kunna få tillbaka sin förmåga till arbete/aktivitet?

☐ Ja, helt ☐ Ja, delvis ☐ Nej

10 Kan resor till och från arbetet med annat färdsätt än det patienten normalt använder göra det möjligt att återgå i arbete? ☐ Ja ☐ Nej

11 Önskar kontakt med Försäkringskassan ☐ Ja ☐ Nej

12 Önskar avstämningsmöte ☐ Ja ☐ Nej

13 Övriga upplysningar

Underskrift

14 Datum (år, mån, dag)

15 Läkarens namnteckning

16 Namn, mottagningens adress, telefonnummer (även riktnr) i klartext (om ej ovan)

Blanketten och mer information finns på www.forsakringskassan.se

72632201

RFV 7263 Formulär fastställt av RFV i samråd med Socialstyrelsen 03.06
