# Supplementary material for: Quality assessment in sickness certificates – changes over an eight-year period in Sweden and associated factors
Source: Scand J Prim Health Care. 2025 Oct 28;44(1):1–13. doi: 10.1080/02813432.2025.2577668 (PMC12918320; doi:10.1080/02813432.2025.2577668)
Supplement: Appendix_4_quality_assessement_protocol_resubmission_1.pdf [file IPRI_A_2577668_SM0704.pdf]

Appendix 4 Quality Assessment Protocol 2012*

Section 1 *Not applicable*

Section 2 Diagnosis

- ☐ Missing
- ☐ ICD-code given
- ☐ Described only in text

Section 3 Present medical history (Choose one or several alternatives)

- ☐ No information given
- ☐ Information given, but sparse and insufficient
- ☐ Information given, but includes irrelevant or inconsistent information
- ☐ Information given. Adequate and comprehensive

Section 4 Functional impairments - observations, findings and results of investigations (Choose one or several alternatives)

- ☐ No information given
- ☐ Information given, but sparse and insufficient
- ☐ Information given, but includes irrelevant or inconsistent information
- ☐ Information given. Adequate and comprehensive

Information is based on

- ☐ No information given
- ☐ Information given

Date of examination

- ☐ No information given
- ☐ Information given

Section 5 Activity limitations based on section 2 and 4 (Choose one or several alternatives)

- ☐ No information given
- ☐ Information given, but sparse and insufficient
- ☐ Information given, but includes irrelevant or inconsistent information
- ☐ Information given. Adequate and comprehensive

There are two versions of section 6 - use the alternative matching the certificate

Section 6 Recommendation – treatment or procedure needed for recovery of work ability

- ☐ No information given
- ☐ Information given

Section 6 a Recommendations

Contact with Swedish Public Employment Service

- ☐ No information given
- ☐ Information given (yes)

Contact with Occupational Health Service

- ☐ No information given
- ☐ Information given (yes)

Other information

- No information given
- Information given (yes) Details:

Section 6b Planned or on-going treatment or procedures

Within health care (explain details below)

- No information given
- Information given (yes) Details:

Other kind of actions needed

- No information given
- Information given Details:

Section 7 Vocational rehabilitation

- No information given
- Information given

Section 8a Work tasks

(Choose one alternative)

- No information given
- Information is not comprehensive
- Information is comprehensive
- Unemployed (yes)
- On parental leave (yes)

Section 8b Degree and length of sick leave

- No information given
- From 201_/ _ _/ _ _ to 201_/ _ _/ _ _ ___ %
- From 201_/ _ _/ _ _ to 201_/ _ _/ _ _ ___ %

Section 9 Comments regarding information given, in relation to Sick-leave guidelines

(choose one or several alternatives)

- No information given
- Information given, but sparse and insufficient
- Information given, but includes irrelevant or inconsistent information
- Information given. Adequate and comprehensive

Section 10 Prognosis

- No information given
- Information given

Section 11 Support needed for travelling to and from work

- No information given
- Information given

Section 12 Contact wanted with the Social Insurance Agency

- No information given
- Information given (yes)

Section 13 Supplementary information

(Choose one or several alternatives)

- Information missing
- Information given, but sparse and insufficient

- ☐ Information given, but lacks relevance or includes inconsistent information, (for example, not explained enough in relation to section 10)
- ☐ Information given is adequate and comprehensive

To what extent does the certificate contain sufficient and relevant information?
(Choose one alternative)

- ☐ A very low extent
- ☐ A rather low extent
- ☐ A rather high extent
- ☐ A very high extent

To what extent does the certificate contain irrelevant or inconsistent information?
(Choose one alternative)

- ☐ Not at all
- ☐ Some extent
- ☐ Rather high extent
- ☐ Very high extent

Assessment of the global quality of the certificate (1-10)
1 Very low global quality
2
3
4
5
6
7
8
9
10 Very high global quality

Note: *English translation by authors
