# Supplementary material for: Quality assessment in sickness certificates – changes over an eight-year period in Sweden and associated factors
Source: Scand J Prim Health Care. 2025 Oct 28;44(1):1–13. doi: 10.1080/02813432.2025.2577668 (PMC12918320; doi:10.1080/02813432.2025.2577668)
Supplement: Appendix 2_Sickness Certificate 2009.pdf [file IPRI_A_2577668_SM0703.pdf]

Appendix 2 Sickness Certificate 2009

Försäkringskassan

Medicinskt underlag för bedömning av förmåga att arbeta vid sjukdom

Klinik eller mottagning, telefonnummer och läkarens namn (om du inte har angett detta längst ner på blanketten)

Patientens namn

Personnummer

Skickas till
Försäkringskassans inläsningscentral

839 88 Östersund

Läkarintyg enligt 3 kap. 8 § lagen (1962:381) om allmän försäkring

Du kan även använda blanketten för avstängning enligt smittskyddslagen (SmL)

Om du inte känner patienten ska hon eller han styrka sin identitet genom legitimation med foto (SOSFS 2005:29)

1

☐ Avstängning enligt SmL på grund av smitta (fortsätt till punkt 8)

Medicinsk bedömning

Vid bedömningen av om sjukdom föreligger ska du bortse från arbetsmarknadsmässiga, ekonomiska, sociala och liknande förhållanden

2 Diagnos/diagnoser för sjukdom/symtom som orsakar nedsatt arbetsförmåga

Diagnosis/diagnoses for disease/symptoms causing limitations in work ability

Diagnoskod enligt ICD-10 (Huvuddiagnos)

Minst tre positioner

3 Anamnes för aktuell sjukdom

Anamnesis for current disease

4 Status och objektiva undersökningsfynd på organnivå (funktionsnedsättning)

Clinical status and objective findings at the organ level (functional impairments)

Jag baserar uppgifterna på

	Datum
☐ min undersökning av patienten	
☐ min telefonkontakt med patienten	
☐ journaluppgifter	
☐ annat (ange vad i punkt 13)	

72632102

5 Hur begränsar sjukdomen patientens förmåga/aktivitet på individnivå? (aktivitetsbegränsning)

How does the disease cause limitations in the patient's ability/activity at an individual level? (activity limitations)

6 Föreskrift - behandling eller åtgärd som är nödvändig för att förmågan ska kunna återställas

☐ Given ordination (ange vilken) ____

☐ Fortsatt poliklinisk kontakt

☐ Undvika viss belastning (ange vilken) ____

☐ Besöka arbetsplatsen

☐ Väntar på åtgärd inom sjukvården (ange vilken) ____

☐ Väntar på annan åtgärd (ange vilken) ____

☐ Övrigt (ange vad) ____

FK 7263 (004 T 001) Fastställd av Försäkringskassan i samråd med Socialstyrelsen 2007-07-09

English translation by the author. Form is published with consent from the Swedish Social Insurance Agency.

7 Är arbetslivsinriktad rehabilitering aktuell?

☐ Ja ☐ Nej ☐ Går inte att bedöma ☐ Patienten behöver få kontakt med företagshälsovård

8 Jag bedömer patientens arbetsförmåga i förhållande till

☐ nuvarande arbete - **ange alltid arbetsuppgifter**

☐ arbetslöshet - att söka och kunna utföra arbete som är normalt förekommande på arbetsmarknaden

☐ föräldraledighet med föräldrapenning - att vårda sitt barn

Jag bedömer att patientens arbetsförmåga är

	från och med (år, månad, dag)	längst till och med (år, månad, dag)
☐ nedsatt med 1/4		
☐ nedsatt med hälften		
☐ nedsatt med 3/4		
☐ helt nedsatt		

9 Jag bedömer att patientens arbetsförmåga är nedsatt längre tid än den som det försäkringsmedicinska beslutsstödet anger, därför att:

72632202

10 Prognos - kommer patienten att få tillbaka sin arbetsförmåga i nuvarande arbete? (Gäller inte arbetslösa)

☐ Ja ☐ Ja, delvis ☐ Nej ☐ Går inte att bedöma (motivera i punkt 13)

11 Kan resor till och från arbetet med annat färdsätt än det patienten normalt använder göra det möjligt för patienten att återgå i arbete? ☐ Ja ☐ Nej

12 Jag vill ha kontakt med Försäkringskassan ☐ Ja ☐ Nej

13 Övriga upplysningar

Underskrift

14 Datum

15 Namnförtydligande, mottagningens adress, telefon (om du inte har angett detta längst upp på blanketten)

16 Läkarens namnteckning

17 Förskrivarkod och arbetsplatskod

FK 7263 (004 T 001) Fastställd av Försäkringskassan
i samråd med Socialstyrelsen 2007-07-09
